# Seroprevalence of human brucellosis in selected sites of Central Oromia, Ethiopia

**DOI:** 10.1371/journal.pone.0269929

**Published:** 2022-12-15

**Authors:** Temesgen Kassa Getahun, Beksisa Urge, Gezahegn Mamo

**Affiliations:** 1 Ethiopian Institute of Agricultural Research Holeta Animal Health Research program, Holeta, Ethiopia; 2 Addis Ababa University College of Veterinary Medicine and Agriculture, Bishoftu, Ethiopia; University of Illinois College of Veterinary Medicine, UNITED STATES

## Abstract

Brucellosis is one of the most neglected zoonotic diseases in the world. It affects all age groups and both sexes. A cross-sectional study was conducted from December 2019 to May 2020 to estimate the seroprevalence and assess the potential risk factors of brucellosis among dairy cow owners and dairy farmworkers, and assess their knowledge, attitudes, and practices in selected sites in the central highlands of Oromia, Ethiopia. A structured interview question was administered to 284 respondents, and only 166 of them volunteered to give a blood sample. Most respondents had limited knowledge of brucellosis (93.3%), zoonotic diseases transmitted by handling animal delivery (88%), and consuming raw milk and other animal products (90.0%). Accordingly, 149 blood samples from animal owners and 17 farmworkers were collected for serological testing. The serum samples collected were initially screened using the Rose Bengal Plate test, and the Complement Fixation test was used as a confirmatory test. The overall seroprevalence of zoonotic brucellosis was 1.2% (95%CI: 0.32–4.27). There was a statistically significant association of human brucellosis with human housing (OR = 1.8, p = 0.002), contact with aborted fetus (OR = 21.19, p = 0.017), drinking raw milk from non-aborted (OR = 24.99, p = 0.012), aborted (OR = 5.72, 0.019), and retained fetal membrane (OR = 4.22, p = 0.029) cows. In conclusion, the present study revealed that the seroprevalence of brucellosis in the study area was low. Public awareness among animal owners, farm and animal health workers on the transmission and health hazards of brucellosis needs to be addressed through community training. Implementing one health approach between veterinary and medical health professionals must be strengthened.

## 1. Introduction

Brucellosis is considered one of the most widespread but neglected zoonotic disease in the world. It is the second most important zoonotic disease in the world after rabies [1]. It is also the most important zoonotic disease in most developing countries, which have no national brucellosis control and eradication program [2].

The genus Brucella is divided into six species based on antigenic/biochemical features and major host species. *B*. *abortus* (cattle), *B*. *melitensis* (sheep and goats), *B*. *suis* (swine, cattle, rodents, and wild ungulates), *B*. *ovis* (sheep), *B*. *canis* (dogs), and *B*. *neotomae* (rodents) are the species responsible. Among the zoonotic pathogens are *B*. *melitensis*, *B*. *abortus*, and *B*. *suis* [1, 3].

It affects people of all age groups and both sexes. In the world 500,000 human infections occur per year, with incidences ranging from less than one case per 100,000 population in the UK, the USA, and Australia, through 20 to 30 cases per 100,000 in southern European countries such as Greece and Spain, to more than 70 cases per 100,000 in the Middle Eastern states such as Kuwait and Saudi Arabia [4, 5]. In some African countries, human brucellosis is expected to be endemic [4].

Human clinical disease is characterized by severe flu-like illness with a high fever that comes and goes (hence the name “undulating fever”), which may progress to a more chronic form with serious complications in the joints (arthritis) or internal organs (heart failure). Humans can become so impaired in this chronic, recurring form that they are unable to work and become a health-care burden on their families [4].

The infection is transmitted by direct or indirect contact with infected animals or their products. A human can acquire brucellosis by contact with infected tissues, blood, urine, vaginal discharges, aborted animal fetuses, and especially placentae. A small number of cases have occurred following accidental self-inoculation of the strain 19 *Brucella* vaccines. It can also be transmitted by inhalation of aerosols, which may occur in animal pens and stables, abattoirs, and laboratories [6].

Several serological studies conducted in Ethiopia over the last two decades have revealed that it is endemic and widespread [7, 8]. The disease is prevalent in cattle and humans in highland and lowland areas [9–14]. Even though a large number of studies on bovine brucellosis have been reported in different parts of the country, studies conducted on the role of bovine brucellosis in relation to public health significance in occupationally exposed individuals are limited in intensive and extensive production systems [15].

There is no information on the seroprevalence of human brucellosis in Holeta Town, in smallholder dairy farms, exposed farm owners and farm employees found in smallholder and government owned farms in Holeta Town, Wolmera District, and Adda Berga Ethiopian Institute of Agricultural Research (EIAR) dairy farm. In addition, assessment of the status of the disease and understanding of the awareness level among the community has paramount importance in order to identify the risk factors for infection and zoonotic transmission and design appropriate measures to reduce the public health significance of brucellosis. Therefore, the objectives of the study were to estimate the seroprevalence of brucellosis in animal owners and farmworkers, to assess the associated risk factors for human brucellosis in the study area, and to assess the knowledge, attitude, and practice of animal owners and farmworkers in relation to zoonotic brucellosis.

## 2. Material and methods

### 2.1. Description of study areas

The study was conducted in Holeta Town, Wolmera District, and Adea Berga EIAR dairy farm, Oromia regional state, Ethiopia, which is known for its well-developed dairy production and constituting the major milk sheds of Addis Ababa, the capital city of Ethiopia.

Holeta, a town in Wolmera District, is situated in the Oromia special zone surrounding the capital city of Addis Ababa. The town is located 29 kilometers west of Addis Ababa at 9°30’ N and 38°30’ E with an altitude ranging from 2300-3800m above sea level, which is actually part of the central highlands of Ethiopia. The average annual minimum and maximum temperatures were 6°C and 22°C, respectively. The annual rainfall ranges from 900–1100 mm. According to the 2007 population and housing census, the population of the town is 23,296 [15, 16].

Adda Berga is a District in Ethiopia’s Oromia Region, located at 9° 15′ 0′′ N, 38° 25′ 0′′ E. It hosts the Ethiopian Institute of Agricultural Research (HARC) dairy farm substation. Adea Berga dairy farm was established at Adea Berga wetland in 1986 for commercial milk production under a government state farm by introducing 400 pure Jersey pregnant heifers and 2 sires (foundation stock) from Denmark [17]. The farm has been engaged in the production and rearing of pure Jersey breeds from the foundation stock for milk supply for dairy development enterprises and also serves as a bull dam station for the national artificial insemination center (NAIC). Then the farm was transferred to the Holeta Agricultural Research Center for a genetic improvement research program in 2007. Currently, this research dairy farm has 350 pure Jersey, Boran, and Holstein Friesian and Jersey cross breeds kept under a semi-intensive rearing system.

### 2.2. Study design and population

A cross-sectional study was conducted from October 2020 to May 2021 to study the seroprevalence of brucellosis. The study included farmworkers from the Holeta and Adda Berga Agricultural Research Center dairy farms, as well as dairy cow owners from Holeta Town and Wolmera District who had direct contact with dairy cows and were willing to participate in the study and sign the informed consent above the age of 18.

### 2.3. Sample size and sampling

A purposive sampling technique was applied to select medium, large and small scale farms based on accessibility and the number of dairy cows owned. The farm owners were also selected purposively based on their milk consumption habits, contact with animals and animal products, and farming system. Accordingly, eight kebeles, eleven medium-scales and one large-scale farm of the Ethiopian Institute of Agricultural Research of HARC from Holeta Town were included. On the other hand, fourteen kebeles out of twenty three kebeles of Wolmera District were selected. The study also included a large-scale farm operated by the Ethiopian Institute of Agricultural Research in the Adda Berga District.

The sample size for the study was calculated using the formula described by [18] with a defined precision of 5% and a 95% level of confidence interval.


$$n=\frac{1.96^{2}\;X\;Pex\;X\;(1-Pex)}{d^{2}}$$

Where, *n =* required sample size, *P*ex = expected prevalence, and *d =* desired absolute precision

Hence, based on the above formula and taking into account 0.05% prevalence, the minimum sample size is:

$$n=\frac{1.96^{2}\;X\;0.05\;X\;(1‐0.05)}{\left(0.05\right)2}$$

n = 380

The estimated sample size was not achieved and only 284 individuals were interviewed, of which 166 (149 voluntary animal owners from Holeta Town and Wolmera District and 17 workers from Holeta and Adda Berga EIAR dairy farms) voluntarily provided blood samples.

### 2.4. Sample collection

#### 2.4.1. Blood sample collection

Blood samples of 5ml were collected from the cephalic vein of study participants by a registered nurse using sterile plain vacutainer tubes. The samples were kept in a slanting position overnight at room temperature to separate the serum according to [3] manual. Then, each serum was gently decanted into sterile screw cupped Eppendorf tubes (1.8ml), labeled, and stored at -20°C in the microbiology laboratory until tested for antibodies using RBPT and CFT for confirmation of the RBPT positive samples. All serum samples were tested for RBPT and CFT in the serology laboratory of the Pathobiology Institute, Addis Ababa, Ethiopia.

### 2.5. Laboratory diagnosis

All serum samples collected from humans were screened using RBPT and CFT (produced by Lillidale diagnostic manufacturer United Kingdom) according to the procedures described by [3, 19] respectively.

### 2.6. Questionnaire survey

A structured interview question was prepared by an animal health team of HARC. All questions were pretested and applied to all concerned workers of the farm and animal owners who have direct contact with animals and animal products in the study area. A format was developed to collect information about personal demography such as age, sex, educational background and knowledge, attitude and practice toward brucellosis, raw milk drinking practice, aborted fetus handling practice, history of abortion, chronic headache, knee pain, and testicular swelling.

The presence of abortions, stillbirths, retention of fetal membranes, a separate parturition/maternity pen in their animal herds, and contact between animals with other herds was recorded. The method of after birth disposal of animals (placenta, aborted material, and dead fetus) was also recorded as burying, eating by dog, or throwing into an open dump.

For the convenience of analysis, each correct response in the knowledge category was scored 1, and each incorrect response was scored 0. The final score was calculated and then labeled based on the score. The correct response to questions >90% was excellent, 75–90% very good, 50–75% good, and <50% poor knowledge [20]. Respondents were asked attitude questions to describe their negative and positive attitudes [20]. For the convenience of analysis, each correct response in the practice category was scored 1, and each incorrect response was scored 0. The responses of respondents were considered good and bad practice [20].

### 2.7. Data management and analysis

Data collected from the field and the serological tests was coded and stored in a Microsoft Office Excel spreadsheet and transferred to R software version 4.0 for statistical analysis. The seroprevalence was calculated on the basis of CFT positivity by dividing the number of positive study participants by the total number of testing humans. The survey data were summarized using frequency and percentage, and Firth’s Bias-Reduced Logistic Regression analysis was used to determine the relationship between seropositivity and potential risk factors [18].

### 2.8. Ethical considerations

The ethical clearance certificate was obtained from the ethical review committee of Oromia Health Bureau based on the assessment of the research proposal on the date of 24/9/2012EC, with ReF. No. BEF/HBTFU/146/914. The standard ethical principles and conducts were implemented in study participants. Written and oral informed consents were obtained from human study participants and livestock owners.

## 3. Result

### 3.1. Demographic characteristics of the respondents

A total of 284 participants were interviewed (213 male and 71 female) to assess their knowledge, attitude, and practices towards brucellosis. Of those respondents, 2 (0.7%) were large-scale farm managers, 11 (3.9%) were medium-scale farm owners, 254 (89.4%) were smallholder farmers, and 17(5.9%) were farmworkers. The majority of the respondent population of the participants (49.3%) was in the 35–45 age groups (Table 1).

**Table 1:** Demographic characteristics of respondents.

Demographic variables	Categories	No. Respondents	Percentage (%)
**Gender**	Male	213	75
	Female	71	25
**Age categories**	<30 years	57	20.1
	31–45 years	140	49.3
	46–60 years	71	25
	>60 years	16	5.6
**Marital status**	Married	242	85.2
	Single	42	14.8
**Educational status**	Illiterate	58	20.4
	Informal	39	13.7
	Non academic	11	3.9
	Primary school	85	29.9
	Secondary school	47	16.5
	College	33	11.7
	University	11	3.9
**Animal ownership status**	Farm employees	17	5.9
	Small holder animal owners	254	89.4
	Medium scale farm owners	11	3.9
	Large scale farm owners	2	0.7

### 3.2. Overall Seroprevalence

**Fig 1:**
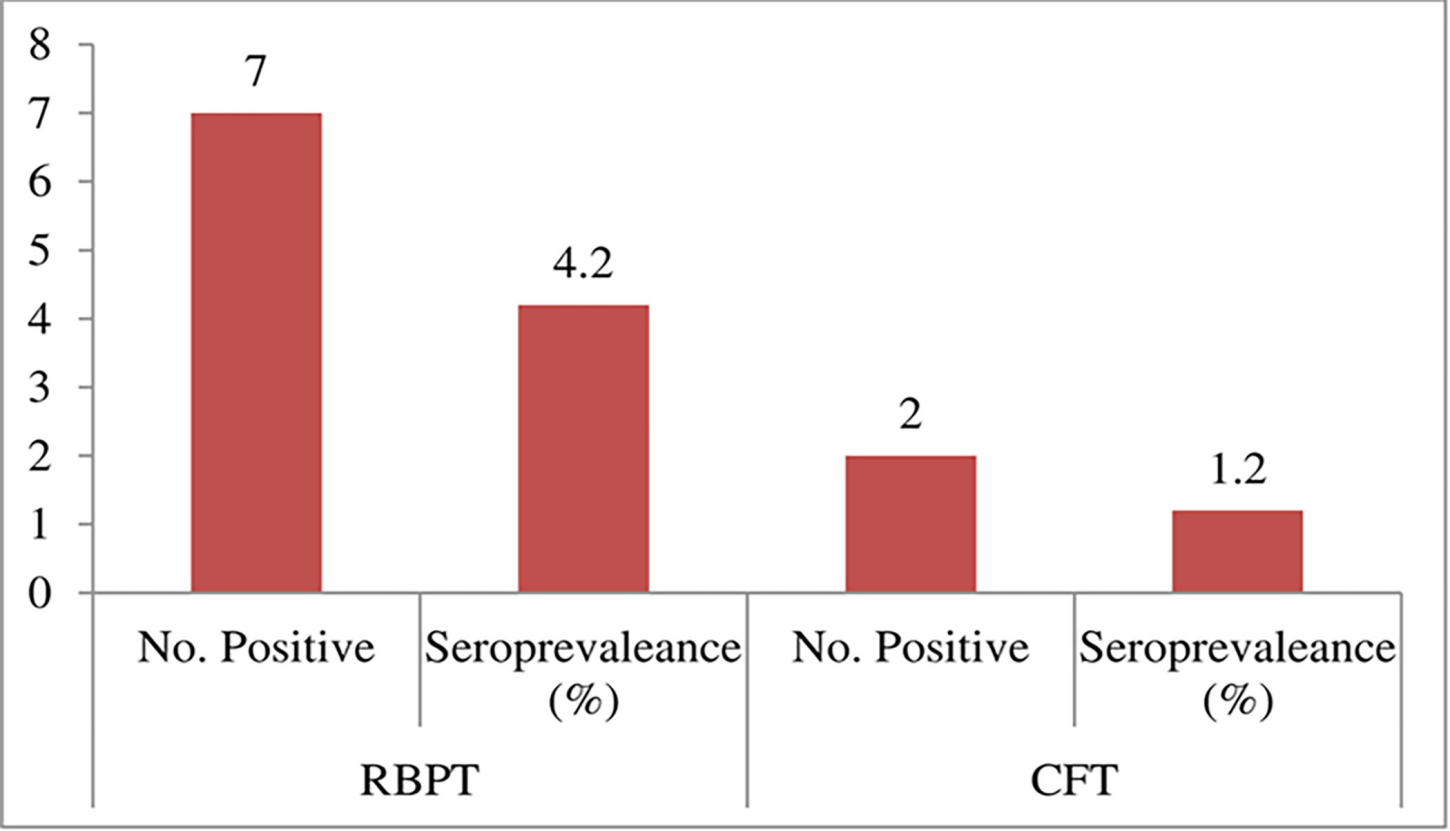
The overall seroprevalence of brucellosis in dairy cattle owners and dairy farm workers with combined RBPT and CFT diagnostic techniques.

The present result revealed that out of 166 human serum samples tested (109 males and 57 females), 7 (4.2%) (95%CI: 2.046–8.425) were tested positive by RBPT and 2 (1.2%) were tested positive by CFT. The overall seroprevalence of human brucellosis among the blood samples tested was 1.2% (95%CI: 0.3289–4.278) in the study area (Fig 1).

### 3.3. Association of putative risk factors

The risk factor analysis revealed statistically significant associations between human brucellosis and being housed with dairy animals (OR = 1.8, p = 0.002), having contact with an aborted fetus (OR = 21.19, p = 0.017), drinking raw milk (OR = 24.99, p = 0.012), drinking raw milk from aborted animals (OR = 5.72, p = 0.019), and drinking raw milk from cows with RFM (OR = 4.217, p = 0.029) (Table 2).

**Table 2:** Univariable Firth’s Bias-Reduced Logistic Regression analysis of risk factors associated with human brucellosis seropositivity.

Variables	Categories	No. sampled	CFT (%)	OR(95%CI)	P-Value
**Origen**	HARC Berga farm	4	0	1.0	
	HARC Holeta farm	13	0	3.3(-1.677–6.601)	0.603
	Holeta Town	55	2(1.2)	4.2(0.276–6.145)	0.623
	Wolmera District	94	0	4.7(-0.243–9.281)	0.191
**Sex**	Male	109	2(1.2)	2.6(0.212–3.713)	0.486
	Female	57	0	1.0	
**Age**	<30 years	41	0	1	
	31–45 years	91	2(1.2)	1.9(0.148–2.661)	0.658
	46–60 years	30	0	0.6(-0.003–0.123)	8.38
	>60 years	4	0	6.9(-0.035–1.377)	0.373
**Educational status**	College	18	0	1.0	
	Illiterate	34	1(0.6)	1.6(0.0839–2.468)	0.752
	Non-academic	4	0	4.1(-0.02–8.131)	0.507
	Informal	6		2.8(-0.014–5.529)	0.616
	Primary school	69	1(0.6)	0.8(0.041–1.202)	0.9004
	Secondary school	28	0	0.6(-0.003–1.223)	0.831
	University	7	0	2.4(-0.012–4.766)	0.663
**Human housing with dairy animals**	Common housing	14	2(1.2)	1.8(0.013–2.426)	0.0022**
	Separate housing	135	0	1.0	
**Contact with aborted fetus**	Yes	89	2(1.2)	21(1.167–2.951)	0.017*
	No	77	0	1.0	
**Condition of contact with aborted material**	Bare hands	89	2(1.2)	1.0	
	Wear gloves	20	0	0.8(-0.602–1.090)	0.912
	Using plastic	55	0	0.3(-0.022–3.854)	0.394
	With other material	2	0	6.9(-0.045–1.258)	0.331
**Washing hands after handling aborted materials**	Yes	147	2(1.2)	1.0	0.609
	No	19	0	0.6(-0.051–9.375)	0. 81
**Consumption of raw milk**	Yes	137	2(1.2)	24(1.196–3.485)	0.012*
	No	29	0		
**Consumption of milk from aborted cow**	Boiled	47	0	1.0	
	Raw	43	2(1.2)	5.7(0.448–7.981)	0.019*
	No consumption	76	0	0.6(-0.0333–1.15)	0.813
**Consumption of milk from RFM cow**	Boiled	75	0	1.0	
	Raw	91	2(1.2)	4.2(0.33–5.852)	0.029*
**History of miscarriage**	Yes	9	0	3.2(-0.023–4.422)	0.51
	No	157	2(1.2)	1.0	
**History of sterility**	Yes	1	0	21(-01359-5.457)	0.167
	No	165	2(1.2)	1.0	
**History of chronic headache**	Yes	66	2(1.2)	7.7(0.621–10.813)	0.117
	No	100	0	1.0	
**History of knee pain**	Yes	68	2(1.2)	7.5(0.605–10.541)	0.123
	No	98	0	1.0	
**History of testicular swelling**	Yes	5	2(1.2)	230(1.0533–3.43)	0.000***
	No	52	0	1.0	

The stepwise multivariable Firth’s Bias-Reduced Logistic Regression analysis results showed important risk factors for brucellosis seropositivity. Generally, there was no multicollinearity between variables. Thus, the analysis revealed that humans who shared housing with dairy animals, had bare-handed contact with an aborted fetus, and drank raw milk were 1.947, 1.022, and 1.019 times more likely to be seropositive to Brucella infection, respectively, than humans who shared separate housing, used protective materials when in contact with an aborted fetus, and boiled before drinking raw milk (Table 3).

**Table 3:** Multivariable Firth’s Bias-Reduced Logistic Regression analysis of risk factors.

Variables	Categories	No. Sampled	CFT (%)	OR (95% CI)	P-Value
**Human housing**	With dairy animals	14	0	1	
	Separate housing	135	2(1.2)	1.9(0.912–0.983)	0.005**
**Contact with aborted fetus**	No	77	0	1	
	Yes	89	2(1.2)	1.02(0.989–1.057)	0.012*
**Consumption of raw milk**	No	29	0		
	Yes	137	2(1.2)	1.01(0.984–1.055)	0.046*

OR = Odds ratio, CI = Confidence interval

### 3.4. Questionnaire surveys

#### 3.4.1. Knowledge, attitude and practices assessment

In the current study, the majority of respondents, 265(93.3%) had poor knowledge of brucellosis, while 19 (6.7%) of the respondents had knowledge of brucellosis in humans and animals. The analysis revealed that the majority of dairy animal owners, 173 (60.9%), consumed raw milk. In the current study, 168 (59.2%) of respondents handled aborted fetuses without using personal protective equipment, while 80 (28.2%) used plastic, 25 (8.8%) used gloves, and the remaining 11 (3.9%) removed aborted fetuses using other materials (Tables 4–6).

**Table 4:** Knowledge of respondents regarding brucellosis.

Variables	Categories	No. Respondents	Frequency (%)	Knowledge level
**Knowledge on Brucellosis**	No	265	93.3	Poor
	Yes	19	6.7	
**Knowledge on zoonotic diseases transmitted by handling of infected animal and animal products**	No	250	88	Poor
	Yes	34	12	
**Knowledge on zoonotic diseases due to raw milk consumption**	No	257	90	Poor
	Yes	27	9.5	

**Table 5:** Attitudes of respondents towards brucellosis.

Variables	Categories	No. Respondents	Frequency (%)	Attitude
**Consumption of raw milk**	No	111	39.1	Positive
	Yes	173	60.9	
**Milk from aborted cow**	Boil before consumption	76	26.8	Negative
	Consume without boiling	80	28.2	
	Not drink	128	45.1	
**Milk from cow with RFM**	Boil before consumption	118	41.5	Positive
	Consume without boiling	166	58.5	

**Table 6:** Practices of respondents regarding brucellosis.

Variables	Categories	No. Respondents	Frequency (%)	Practice
**Contact with aborted fetus**	No	116	40.8	Bad
	Yes	168	59.2	
**Condition of contact with aborted material**	Bare hands	168	59.2	Bad
	Wear gloves	25	8.8	
	Wear plastic	80	28.2	
	With other material	11	3.9	
**Hand washing after contact with aborted fetus**	No	259	91.2	Bad
	Yes	25	8.8	

## 4. Discussion

Serum samples from 149 recently aborted dairy cow owners and 17 farm workers were examined for the presence of brucellosis. Of the total examined humans, the seroprevalence of brucellosis using RBPT and CFT was found to be 4.2% and 1.2%, respectively. Since CFT is recommended as a confirmatory test for brucellosis with high specificity [21, 22], the overall seroprevalence of human brucellosis in the study area was 1.2%. The findings of this study are relevant to the country’s development of a national brucellosis control program by the medical and veterinary sectors. Our study provides evidence that brucellosis is one of the public health problems among the rural and urban population of Ethiopia [23].

In Ethiopia, very few studies have been conducted to determine the seroprevalence of human brucellosis. Similar studies by [24] (1.2%) in Western Tigray; [25] (1.2%); [10] 1.3% in Debrezeit and Mojo were reported. Compared to the present study, however, the very higher sero-prevalence of brucellosis observed by [26] (12.5%); [27] (3.4%); [28] (4.8%); [29] (3.8%); [9] (16.5%); [24] (2.2%); [25] (2.2%) [30], 5.8% and 9%, and [31] 11% might be attributed to the large sample size involved and/or the different confirmatory tests used by the two studies, the CFT versus the 2–mercaptoethanol test (MET). The lower prevalence in our study could also be due to the fact that the number of livestock, level of contact with animals, and frequency of consumption of dairy products are low as compared to the mentioned study. Rather, very high *Brucella* infection was reported in abattoir workers in other countries [32, 33]. They reported a prevalence of 19.69% and 21.7% among slaughterhouse workers in India and Pakistan, respectively. This might be correlated with the exposure of an abattoir worker to brucellosis infected animals and animal discharges. Brucellosis is an occupational disease, occurring most often in veterinarians, farmers, stock inspectors, abattoir workers, laboratory personnel, and butchers [34].

Statistically significant associations between human Brucella seroprevalence and human housing and contact with aborted fetuses were agreed with reports from Ethiopia [9, 26] and another country [35]. This may be due to more cases of human brucellosis occurring in rural areas where most of the people are farmers or in close contact with animals. The other possible explanation that could be given from this finding is that both farmers, animal health personnel, and farm government employees could be infected while helping infected cows during parturition, either through abrasions or the conjunctiva, acquire infections by handling tissues containing *Brucella* organisms, and also contract brucellosis either by handling infected animals or by living with infected animals in similar houses.

In the present study, a strong association was observed between human brucellosis and handling of parturient materials, drinking raw milk, drinking raw milk from aborted animals, and drinking raw milk from cows with RFM, which is in agreement with studies conducted by [36, 37]. The possible reason for this may be that in our study areas most of the participants drank raw milk, including raw milk from aborted and retained fetal membrane cows as it was assessed with interview questions. The primary methods of transmission of *Brucella* are through raw milk and contact with aborted materials.

The clinical manifestations pertaining to human brucellosis found in this study were similar to previous findings reported by [9, 36, 38]. Apart from general clinical manifestations, participants with a history of testicular swelling had a statistically significant association with *Brucella* seroprevalence. This might be due to the fact that the primary manifestation of brucellosis in man is a chronic headache at the early stage and swelling of the testicle due to Orchitis.

There was no statistically significant association between the sexes, even though a high prevalence was seen in males. This finding was in agreement with [38, 39]. All the positive sera were from the male participants. This may reflect cultural and social behavior patterns whereby males are actively involved in caring for domestic animals in central highland areas. In this study, a higher proportion of seropositivity was observed in the age category of 31–45 years of age, although the different age categories did not differ significantly. This finding was agreed with that of [28, 38, 40]. This difference might have been associated with the fact that in the study area, age categories that were between 31–45 years of age were responsible for handling aborted animals, milking of cows, and continuous contact with animals.

A total of 267 cattle owners and 17 government farm workers were interviewed to assess their awareness levels regarding animal management, brucellosis and occupational risks using structured interview questions. Knowledge of diseases is a crucial step in the development of prevention and control measures [36]. Despite the huge efforts of the government and non-government institutions to improve animal production in the areas, general knowledge of brucellosis among the farmers was still poor. The educational status attained by a majority of the respondents was low, which falls between reading and writing and lower grades. In addition to this, barn hygiene, proper disposal of aborted materials, and the use of a separate parturition pen were not well cooperated with, especially by smallholder farmers. This could have led to high risks of transmitting the disease within and between the herds and humans. This is in agreement with previous studies of [41–43]. Likewise, mixing of different animal species has its own economic importance in increasing the chances of transmission of brucellosis to cattle.

Concerning the knowledge of brucellosis and other zoonotic diseases, interview question based data were collected. The overall awareness level of brucellosis and other zoonotic diseases among smallholder farmers, medium scale farm owners, and farm workers were found to be relatively low. In addition, most of them were not wearing protective gloves or other materials while handling aborted animals and aborted materials. A similar finding was also reported by [10]. This might be attributed to their educational status, since most of the farmers could not read and write or have a primary education background. Additionally, it could also be due to a lack of awareness creation programs about zoonotic diseases. Generally, a lack of sufficient knowledge of brucellosis and other zoonotic diseases, unprotected working conditions, regular exposure from aerosol and contact through cuts and abrasion to infected materials such as aborted materials, carcasses, viscera, organs, blood and urine are considered fertile grounds for exposure and transmission of the diseases to humans. In this regard, very little has been done by way of awareness creation about brucellosis.

The result of the knowledge assessment showed that brucellosis was not well-known by the general community in the present study area, since around 93.3% of the study respondents had no information about brucellosis. This is similar to findings of previous studies done in Kenya, where 80% of respondents knew nothing of the existence of brucellosis, whereas in Tajikistan 85% of the respondents had never heard of brucellosis [44].

Most of the respondents from small holder dairy farms did not understand the methods of zoonotic disease transmission, including brucellosis. Farmers’ lack of awareness about brucellosis, improper handling of aborted materials, and the habit of consuming raw milk, among other factors, might contribute to the further spread of brucellosis in their livestock and expose the community to a public health hazard [42]. This low awareness is a limiting factor if control strategies are to be implemented. Lack of knowledge of the causative agent, mode of transmission, and preventative measures against brucellosis can be detrimental. It is therefore important to establish an educational campaign in the study areas to enlighten the communities on the disease, risk factors, as well as control strategies, particularly for both livestock and humans.

Based on the questionnaire survey, most of the respondents handled abortion and placental retention without using personal protective equipment (59.2%); even though using personal protective equipment is the most known measure against zoonotic brucellosis. These factors combined with the poor hand cleaning practice after contact with aborted materials (91.2%) by the owners could pose a great risk of the spread of the disease to unaffected animals [27]. Furthermore, in this study, the majority of respondents (79.2%) did not bury the afterbirth (aborted fetus, stillbirth, and retained fetal membrane), but rather left them on an open field.

## 5. Conclusion and recommendations

The present study revealed that the overall seroprevalence of human brucellosis on animal owners and farm workers was 1.2% in Holeta Towns, Wolmera District, and Adea Berga EIAR dairy farm in Oromia Regional State, Ethiopia. The finding of positive serological reactors did not only suggest the presence of the disease in the population in the areas, but also indicated the presence of foci of infection that could serve as sources of infection for the spread of the disease into unaffected humans. In this finding, human housing, contact with an aborted fetus and RFM, drinking raw milk, drinking raw milk from aborted animals, and drinking raw milk from cows with RFM were statistically important risk factors associated with human brucellosis seropositivity. This study also provided important information on the knowledge, attitude, and practice of livestock owners and occupational workers about brucellosis that result in the significant zoonotic importance of using raw milk for human consumption. This emphasizes the impact of brucellosis on public health and the need to control and prevent brucellosis in the study areas.

Based on the above conclusions, the following recommendations are forwarded to curb the further spread of the disease in human populations: Public awareness among animal owners, butchery men, abattoir workers, and animal health workers on the transmission and health hazard of brucellosis needs to be addressed through community training, aborted animals must be isolated, aborted fetuses and fetal membranes must be disposed of properly, preferably by incineration, further research into the isolation and identification of Brucella biotypes involved at the livestock-human interface could be beneficial.

## Supporting information

S1 File (DOCX)

S2 File (DOCX)

S3 File (DOCX)
