# Supplementary material for: Seroprevalence of human brucellosis in selected sites of Central Oromia, Ethiopia
Source: PLoS One. 2022 Dec 15;17(12):e0269929. doi: 10.1371/journal.pone.0269929 (PMC9754185; doi:10.1371/journal.pone.0269929)
Supplement: S1 File — (DOCX) [file pone.0269929.s001.docx]

Ethical clearance certificate
