# Supplementary material for: Seroprevalence of human brucellosis in selected sites of Central Oromia, Ethiopia
Source: PLoS One. 2022 Dec 15;17(12):e0269929. doi: 10.1371/journal.pone.0269929 (PMC9754185; doi:10.1371/journal.pone.0269929)
Supplement: S2 File — (DOCX) [file pone.0269929.s002.docx]

Interview Question

Seroprevalence and isolation of *brucella* species from cattle and its public health significance in holeta and its surrounding, Oromia region, Ethiopia

Date_______/_________/_________

Code ____________________

Enumerator Name ____________________________

I, General information

1. Name of respondent______________________________________

1.1. Age: ______________1.2. Sex: _______________1.3 Address--------------------------

1.4. Educational background a) Primary school, c) College/University,

b) Secondary school, d) nonacademic/informal, e) Illiterate

**II. Husbandry Practice**

1. Type of housing a) separate, b) common

2. Human housing

A) Housed with family and livestock

B) Housed in family without livestock

3. General Hygiene of the House: A) Very good, B) Good C), Satisfactory D) Poor

4. Do you separate cows during parturition? Yes/ No

5. What are the commonly encountered disease affecting cattle in order of importance?

A._________________________ C._______________________

B__________________________ D________________________

6. Do you know disease that causes abortion in cattle? Yes/No

7. What are the names of the disease locally?

A)._______________________________

B)__________________________________

C)__________________________________

8. Was any event of abortion, still birth and retained fetal membrane in your farm? yes/no

| Problems | Age | Time since it occur |
| --- | --- | --- |
| Abortion |  |  |
| Still birth |  |  |
| Retained fetal membrane |  |  |

11. Do you know disease Brucellosis A) yes, B) no

10. Do you have direct contact with aborted/still birth/RFM?

11. If yes for Q10, how do you make a contact? A) Wear glove, B) Wear plastic, C) Bare hand

12. If yes for Q10, do you wash your hand? A) Yes, B) No

13. Where do you dispose the aborted/still birth/RFM birth or retain placenta?

A) Burying B) Open dump C) Fed to Dogs D) Through over the field, E) Other______________

14. How do you dispose the aborted fetus? A) Using protective B) Bare hand

15. What are the reasons of culling in the farm? A) Disease, B) Old age, C) Infertility,

D) Poor production, E) Others

16. Do you know any disease transmitted from animal to human through handling of infected animals and its products? a. Yes b. No _______________________________________________

17. Do you know any zoonotic diseases that transmit through milk consumption a. Yes b. No

______________________________________________________________________________

18. Do you know any diseases that transmit during handling of delivery or abortion? a. Yes b. No

_____________________________________________________________________________

19. Have the farm/ herd been tested for brucellosis? a. Yes b. No. When____________________

20. What do you do with the known *Brucella* infected Animals? A). Segregation, A) Left with herd, B) Slaughter, D. Both

21. What do you do to the calving pen after parturition? A) Flushing with water, B) Disinfecting with detergents, C) Both, D,non

22. What do you do for the milk from aborted/still birth caw? A) Drink without boiling, B) Boiling, C) Did not drink

Annex 5: Format used for individual human sampling

| No/ | Owner Name | Age | Sex | Abortion | Chronic headache | Orchitis | Knee pain | Sample type | Test type | Result |
| --- | --- | --- | --- | --- | --- | --- | --- | --- | --- | --- |
|  |  |  |  |  |  |  |  |  |  |  |
|  |  |  |  |  |  |  |  |  |  |  |
|  |  |  |  |  |  |  |  |  |  |  |
|  |  |  |  |  |  |  |  |  |  |  |
|  |  |  |  |  |  |  |  |  |  |  |
|  |  |  |  |  |  |  |  |  |  |  |
|  |  |  |  |  |  |  |  |  |  |  |
|  |  |  |  |  |  |  |  |  |  |  |
|  |  |  |  |  |  |  |  |  |  |  |
|  |  |  |  |  |  |  |  |  |  |  |
|  |  |  |  |  |  |  |  |  |  |  |
|  |  |  |  |  |  |  |  |  |  |  |
|  |  |  |  |  |  |  |  |  |  |  |
|  |  |  |  |  |  |  |  |  |  |  |
|  |  |  |  |  |  |  |  |  |  |  |
|  |  |  |  |  |  |  |  |  |  |  |
