# Supplementary material for: Seroprevalence of human brucellosis in selected sites of Central Oromia, Ethiopia
Source: PLoS One. 2022 Dec 15;17(12):e0269929. doi: 10.1371/journal.pone.0269929 (PMC9754185; doi:10.1371/journal.pone.0269929)
Supplement: S3 File — (DOCX) [file pone.0269929.s003.docx]

Laboratory protocols

1. Rose Bengal Plate Test Procedures

Reagents:

- RBT *Brucella* antigen
- Positive control sera (from previously positive serum)
- Negative control sera (from previously negative serum)
- Test sera

Materials:

- Plate
- Micro pipette of 30 μl
- Micro pipette tips
- Applicator
- Magnifying glass
- Tube of serum collection
- Vacutainer tubes fitted with handle and needles
- Rack

Procedure

1. Sera (control and test sera) and antigen for use were left at room temperature for half an hour before testing; since active materials straight from the refrigerator react poorly. 30μl serum was mixed with 30 μl volume of antigen on a white tile or enamel plate to produce a zone approximately 2 cm in diameter.
2. The antigen and serum were mixed thoroughly using an applicator stick (a stick being used only once).
3. Rock plate by hand for about 4 minutes.
4. Examine for agglutination in a good light.
5. Use magnifying glass when micro agglutination suspected.

Interpretation:

0 = no agglutination

+ = barely perceptible

++ = fine agglutination, some clearing

+++ = coarse clumping, definite clearing

2: Complement Fixation Test Procedures

In the CFT, all reagents were evaluated by titration. The preparation of sheep red blood cells (SRBC), the methods of CFT test, and preparation of reagents were according to the protocol of BgVV Service Laboratory (2000).

Reagents:

- Veronal buffered diluent (prepared by mixing 1vial of the constituents into 1 liter of distilled water (PH 7.25).
- Working strength antigen
- Complement
- Ambocepter
- Alsever’s solution
- Positive control sera
- Negative control sera (from previously tested

Materials:

- Micro titer plate (U-shaped)
- Shaker housed in an incubator (37 ºC)
- 0-100 μl adjustable single channel pipette
- 25 μl multi-channel pipette
- 1000 μl adjustable single channel pipette
- Pipette tips
- Plastic troughs
- Traditional glass pipette
- Centrifuge tubes
- Measuring cylinders
- Glass and plastic beakers
- Water bath (58 ºC)
- Centrifuge
- Refrigerator
- Rack

i. Preparation of SRBC for hemolytic system

10 ml of SRBC in Alsever’s solution were centrifuged at 2500 rpm for 5 minutes. The supernatant was discarded and replaced by veronal buffer diluents (VBD). The SRBC were re-suspended in the diluent and centrifuged again. This procedure was repeated 4 times. Before discarding the supernatant after the last washing, the packed cells volume was measured. The volume of the packed cells was read by placing an identical tube next to the blood containing tube and filled up to the level of the blood by a measured amount of water. Finally, a 2% suspension of SRBC was prepared.

ii. Amboceptor titration

1. Two rows of 5 test tubes each were arranged on a rack
2. In two other test tubes, 1: 500 and 1: 750 prediluted were mad
3. 1ml of 1: 500 prediluted amboceptor was transferred to the first test tube of row 1 and 1 ml of 1: 750 prediluted amboceptor was transferred to the first test tube of row 2
4. 0.5ml VBD was added to each of the rest of tubes of both rows
5. Amboceptor was then diluted serially from tube 1 to tube 5 in 0.5 ml amount in both rows. Thus the dilution ran from 1: 500 to 1: 8000 and 1: 750 to 1: 12000 in row 1 and row 2, respectively
6. To each tube of the two rows, 1ml of VBD was added
7. Following, 0.5 ml of 2 % SRBC was added to each test tubes of the two rows and were shacked well
8. The tube were left on the table for 10 minutes
9. 1 ml of complement at working dilution was added and incubated at 37 0C for 30 minutes
10. The last tube showing complete hemolysis, minimum hemolytic dose (MHD) was read. The working dilution of amboceptor is 4 times MHD (BgVV Service Laboratory, 2000)

iii. Evaluation of complement

1. Freeze dried complement was reconstituted according to its instructions
2. A 1: 100 complement dilution was prepared
3. Complement was added into 9 wells increasing by 5μl every time, starting with 10μl
4. Diluent was added into the 9 wells in decreasing amounts by 5 μl , starting with 40 μl
5. 25μl of a diluent was added into the wells with Cornwall syringe
6. The plate was placed in water bath at 37^0^C for 1 hour
7. 25μl 2 % SRBC was added to all wells
8. 25μl amboceptor at working dilution 1:1000 was
9. added to all wells
10. Components were mixed by shaking and incubated again in water bath at 37 oC for 30 minutes

The test was read by recording minimum hemolytic dose of complement (MHD) which was represented by the first well showing complete hemolysis. The next well contains the full hemolytic dose (FHD). The working dilution of complement was then computed: complement dilution= 2FHD/ initial dilution of complement.

iv. Antigen titration

Micro titer plate I:

1. 25μl of VBD was first placed to every walls of a micro titer plate
2. 25μl of a pre diluted antigen was added to all wells of row A
3. By serial doubling (two fold ) dilution 25μl of antigen was transferred from row A to B and from row B to C until row G by multi-channel pipette; 25μl mixture was discarded from row G

Micro titer plate II

1. 50μl VBD was added in all wells
2. 50μl of prediluted inactivated positive control serum was added to all wells of column 1
3. 50μl was serially transferred by two fold dilution, from column 1to column 2, and again from column 2 to column 3, until column 11 from where 25 μl was discarded (column 12 had only VBD)

Mix plate I and II

1. 25μl was transferred from plate II to plate I
2. 25μl of complement in at working dilution (1:40) was added to all wells of plat I
3. Plate I was incubated 37^0^C for 30 minutes (sealed)(warm fixation)
4. The following, 25μl of equal volumes of 2% SRBC and amboceptor ( working dilution) pre-mixed were added to all wells
5. The plates were covered with sealing tape and shacked placed in an incubator (37^o^C) for 30 minutes (warm fixation)

The interpretation was performed as follow:

The last wells with 50% sedimentation was read and recorded. This was regarded as the right corner value. In this case, the corner value was 1: 25 dilution and was used throughout the test.

The 50% sedimentation was taken as one unit and the working dilution of the antigen was two units.

Test procedure:

1. The sera were pre diluted at 1:2.5 and incubated at 58^o^C in a water bath for 30 minutes in order to inactivate the native complement
2. 25μl of diluted test sera was placed in wells of first and second rows of U-bottom plate, and 25μl of veronal buffer was added to all wells except those of the first row
3. Serial doubling dilution were then made by transferring 25μl volumes of serum from 2nd row on wards continuing for at least four dilution
4. 25μl of antigen diluted to working dilution excluding those of anticomplemetary controls, which received 25μl VBD was added to all wells
5. 25μl of complement (1: 40 working dilution) in working dilution was added to all wells except control wells
6. Control wells containing: serum control has serum + complement + diluent + and antigen control has antigen + complement + diluent. Complement control has complement + diluent and hemolytic system has diluent set up to contain 75μl total volume in each case before hemolytic system was added
7. The plates were incubated for 30 minutes at 37 ^0^C with agitations (warm fixation)
8. 25μl of 2 % SRBC and amboceptor (hemolytic system) mixture was added into all the wells
9. Plates were sealed with sealing tape and placed on a shaker and incubator (37 ^0^C) for 30 minutes
10. Before reading the result the plates were left in the refrigerator at +4^o^C for one hour in order to allow non lysed cells to settle
11. Plates were taken out from refrigerator and results were read after being left on the table for 10 minutes at room temperature
12. Positive reactions were indicated by the absence of hemolysis, sedimentation of SRBC, and negative reactions by the hemolysis of SRBC

The interpretation was performed as follow:

Sera with at least 50% fixation of the complement at a dilution of 1:10 were taken as positive. A hemolytic reaction of 50% or less at a dilution of 1:5 was considered as the minimum sero-positive threshold (Dohoo *et al*., 1985).
